# Chronic Liver Disease: Assessing Inflammation and Fibrosis Using Three‐Dimensional MR Elastography With Same‐Day Biopsy in a Prospective Cohort

**DOI:** 10.1002/jmri.70319

**Published:** 2026-04-02

**Authors:** Shan Cai, Christian Simonsson, Markus Karlsson, Wile Balkhed, Jens Tellman, Simone Ignatova, Patrik Nasr, Mattias Ekstedt, Stergios Kechagias, Wolf C. Bartholomä, Nils Dahlström, Peter Lundberg

**Affiliations:** ^1^ Department of Health, Medicine and Caring Sciences Linköping University Linköping Sweden; ^2^ Center for Medical Image Science and Visualization (CMIV) Linköping University Linköping Sweden; ^3^ Department of Biomedical Engineering Linköping University Linköping Sweden; ^4^ Clinical Department of Gastroenterology and Hepatology Region Östergötland Linköping Sweden; ^5^ Clinical Department of Medical Radiation Physics Region Östergötland Linköping Sweden; ^6^ Department of Clinical Pathology Region Östergötland Linköping Sweden; ^7^ Wallenberg Center for Molecular Medicine (WCMM) Linköping University Linköping Sweden; ^8^ Clinical Department of Radiology in Linköping Region Östergötland Linköping Sweden

**Keywords:** chronic liver disease, fibrosis, inflammation, MR elastography, steatosis

## Abstract

**Background:**

Three‐dimensional (3D) MR elastography (MRE) derives viscoelastic parameters that may reflect inflammation, but their frequency dependence and the influence of steatosis on inflammation grading and fibrosis staging remain unclear.

**Purpose:**

To investigate 3D multifrequency MRE for assessing hepatic inflammation, fibrosis stage across frequencies, and the influence of steatosis.

**Study Type:**

Prospective.

**Population:**

Sixty‐four (40 men, median age: 58 years) participants with chronic liver disease (CLD); 21 (8 men, median age: 28 years) healthy volunteers.

**Field Strength/Sequence:**

3‐T; gradient‐echo sequence with mechanical vibrations at low (16.7 and 18 Hz), medium (33.4 and 36 Hz), and high (50.1 and 54 Hz) frequencies.

**Assessment:**

In CLD participants, MRE‐derived viscoelastic parameters, shear stiffness, storage modulus, loss modulus, and damping ratio were compared with histologically assessed fibrosis, inflammation, and steatosis. MRE test–retest repeatability over 10 min was evaluated in healthy volunteers.

**Statistical Tests:**

Wilcoxon rank sum test, Spearman's correlation, multivariable regression analysis, and area under the receiver operating curve (AUROC). A *p* value of < 0.05 was considered statistically significant.

**Results:**

Inflammation was significantly independently associated with damping ratio at medium frequency, which showed moderate performance for grading inflammation (AUROC = 0.76–0.83, sensitivity = 0.83–0.84, specificity = 0.70–0.79). Fibrosis staging using shear stiffness and moduli showed high diagnostic performance (AUROC = 0.82–0.95), with comparable accuracy between medium and high frequencies (*p =* 0.327–0.896). Steatosis was not significantly correlated with MRE overall (*p* = 0.212–0.459), but was significantly associated with 19% higher stiffness and 20% higher loss modulus at medium frequency in CLD participants without fibrosis or inflammation.

**Data Conclusion:**

Medium frequency 3D MRE demonstrated an independent association with inflammation while preserving accurate fibrosis assessment. Steatosis seemed not to confound MRE‐based evaluation.

**Level of Evidence:**

1.

**Technical Efficacy:**

Stage 2.

## Plain Language Summary

Chronic liver disease can cause both inflammation and scarring (fibrosis). Accurate assessment usually requires a biopsy, which is invasive. This study evaluated a noninvasive imaging method called three‐dimensional magnetic resonance elastography (3D MRE) in patients who underwent same‐day liver biopsy. The researchers tested whether different vibration frequencies could detect inflammation and fibrosis. They found that medium frequency measurements were associated with liver inflammation while still accurately identifying fibrosis. Fat accumulation in the liver did not significantly affect the results. These findings suggest that 3D MRE may help medical doctors assess liver inflammation and fibrosis noninvasively in a single examination.

## 1 Introduction

Chronic liver disease (CLD) can progress from chronic inflammation to fibrosis and ultimately cirrhosis [1, 2]. Early detection of inflammatory activity and fibrotic remodeling is important for preventing the progression to irreversible fibrosis. Although liver biopsy remains the reference standard, it is invasive and limited by sampling errors and inter‐ and intra‐observer variability [3, 4]. Noninvasive alternatives such as serum biomarkers and imaging techniques have therefore been proposed [5].

Among these, magnetic resonance elastography (MRE) has proved the most accurate for assessing liver fibrosis [6, 7, 8]. However, conventional two‐dimensional (2D) MRE cannot determine whether increased stiffness is caused by liver fibrosis or inflammation. Three‐dimensional (3D) MRE addresses this limitation by estimating the viscoelastic properties, including the shear stiffness (|G*|), storage modulus (*G′*), loss modulus (*G″*), and damping ratio (*ζ*) [9], that may allow differentiation of fibrosis from inflammation. Recent preclinical and early clinical studies have shown the potential of viscosity‐related parameters, such as the loss modulus and damping ratio, for assessing inflammation [10, 11, 12, 13, 14, 15].

Despite these promising findings, the diagnostic utility of 3D MRE for grading inflammation remains underdeveloped, and several questions warrant further investigation: the sensitivity of viscoelastic parameters to histopathological features may vary at different frequencies [9, 10], and the influence of hepatic steatosis on these viscoelastic parameters remains inconclusive [15, 16, 17, 18].

Therefore, the present study aimed to prospectively investigate the role of 3D MRE‐derived viscoelastic parameters at multiple frequencies in assessing hepatic inflammation in patients with CLD. Secondary aims were to compare the diagnostic performance of 3D MRE for fibrosis staging at different frequencies and to explore the influence of steatosis on viscoelastic parameters.

## 2 Materials and Methods

### 2.1 Study Design and Participants

**FIGURE 1:**
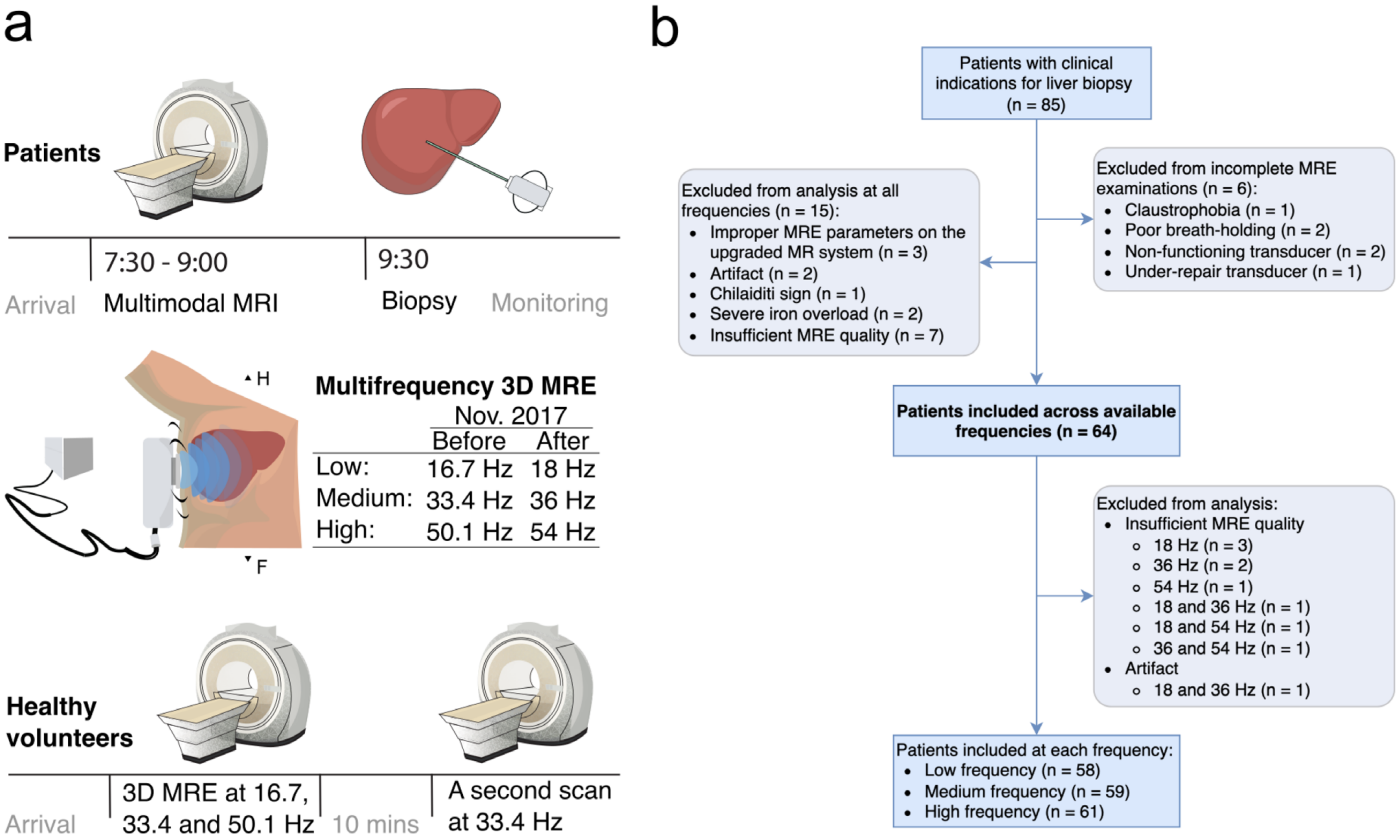
Study design and flow chart of CLD participants. (a) Study design. CLD participants arrived in the morning on an outpatient basis. They underwent a multiparametric MRI examination, including 3D MRE at three driving frequencies: Low (16.7 and 18 Hz before and after November 2017, respectively), medium (33.4 and 36 Hz before and after November 2017, respectively), and high (50.1 and 54 Hz before and after November 2017, respectively) frequencies. Within 1 h following the MRI examination, CLD participants underwent an ultrasound‐guided clinical liver biopsy and were monitored for any complications. Healthy volunteers were also recruited to assess the MRE repeatability with repositioning (10‐min interval). (b) A flowchart shows CLD participants excluded from the analysis. H, head; F, feet.

The study was approved by the Regional Ethical Review Board (Dnr 2015/14–31). Written informed consent was obtained from all participants. Between 2015 and 2022, 85 participants with suspected CLD with clinical indications for liver biopsy and with eligibility for MRI examinations were consecutively recruited from the Department of Gastroenterology and Hepatology at the University Hospital in Linköping, Sweden. CLD participants underwent an extensive MRI examination, including multifrequency 3D MRE, in conjunction with the liver biopsy, which was performed within 1 h of the examination (Figure 1a). A separate cohort of 21 healthy volunteers, with no self‐reported liver disease and no contraindications to MRI, was recruited in 2015 to assess the repeatability of MRE and serve as a reference for quality control of CLD participants' MRE measurements.

### 2.2 3D MRE Acquisition

Participants fasted for 3 h before the MRI examination. The 3D MRE was performed on a 3 T Ingenia MR‐scanner (Philips Healthcare, Best, The Netherlands) using an abdominal surface coil array. Mechanical waves were generated with an electrodynamical transducer (Philips Healthcare, Hamburg, Germany) placed on the right rib cage of the participant in the supine position. Participants with CLD were scanned using one of two MRE protocols: Protocol 1 before a system upgrade in November 2017, with low, medium, and high vibration frequencies of 16.7, 33.4, and 50.1 Hz; and Protocol 2 after the upgrade, with frequencies of 18, 36, and 54 Hz. MRE repeatability was assessed in the healthy volunteers using Protocol 1. After completion of the Protocol 1 scan, the healthy volunteers were instructed to stand for 10 min before being repositioned for a second scan at 33.4 Hz. Images were acquired in four breathholds using a gradient echo‐based sequence, eXpresso [19], with fractional encoding. Main MRE acquisition parameters: field‐of‐view = 320 × 320 mm^2^, matrix size = 80 × 80, slice thickness = 4 mm, # slices = 8/9 and flip angle = 15°. Detailed acquisition parameters are presented in Table S1.

### 2.3 Image Analysis

3D MRE reconstruction was performed using a custom software package, KIR (King's College, London, UK) [20]. Parametric maps of shear stiffness (|G*|), storage modulus (*G′*), loss modulus (*G″*), and damping ratio (*ζ* = *G″/2G′*) were generated. Regions of interest (ROIs) were drawn near the placement of the transducer, avoiding the liver borders and large blood vessels, on the MRE magnitude images by a biomedical engineer (S.C.) with 2 years of experience in abdominal imaging under supervision from a radiologist (N.D.) with 20 years of experience in abdominal imaging, who were blinded to the histological results. For each participant, an ROI was first placed in the high frequency‐acquired image and then copied to the other frequencies, with manual adjustment when necessary.

To ensure sufficient MRE data quality, pixels with a curl‐over‐divergence ratio below two or nonlinearity exceeding 50% were filtered out within each ROI [21]. Mean values of |G*|, *G′*, *G″*, and *ζ* were calculated within the ROI after filtering, using central slices with the top two and bottom two slices excluded to avoid potential boundary artifacts. Datasets with a filtered ROI volume below 13 cm^3^ were considered insufficient in quality and excluded from further analysis [22].

Additional quality assessment included evaluation for severe artifacts, severe iron overload (evident as markedly low signal intensity on the MRE magnitude images), and the presence of the Chilaiditi sign, which can obstruct wave transmission into the liver. Image quality was assessed by a radiologist (N.D.) with 20 years of experience in abdominal imaging.

Inter‐observer reproducibility was evaluated in the CLD participants included in the final analysis. A second radiologist (W.C.B) with 16 years of experience in abdominal imaging independently placed the ROIs, blinded to the first observer's results. The reported measurements were obtained after applying the same quality‐filtering criteria. All image post‐processing was performed using MATLAB (R2024a, MathWorks Inc., Natick, MA, USA).

### 2.4 Liver Biopsy and Histological Evaluation

Liver biopsy was performed within 1 h after the MR acquisition using an ultrasound‐guided 16‐gauge (1.6‐mm) needle (BioPince Full Core Biopsy Instrument; Argon Medical Devices, Plano, TX). All biopsy samples were evaluated by a liver pathologist (S.I.) with 26 years of experience, who was blinded to the clinical data and MRE results. Hepatic fibrosis stage and inflammation grade were assessed using the Ishak scoring system, which comprises an inflammation scale (0–18) and a fibrosis scale (0–6) [23]. Hepatic steatosis was graded on a four‐point scale based on the fraction of fat deposition in hepatocytes using the Kleiner system [24].

### 2.5 Statistical Analysis

Participants with incomplete or technically inadequate MRE examinations, or with missing or non‐diagnostic biopsy were excluded from subsequent analyses. Continuous variables are presented as means with standard deviations (SDs) or medians with interquartile ranges (IQRs), when appropriate, and categorical variables are presented as counts with percentages. The normality of the continuous variables was assessed using the Shapiro–Wilk test.

The 3D MRE repeatability was assessed using the percentage repeatability coefficients (%RC) [22], calculated as $1.96\times \sqrt{2\times \%{\mathrm{wCV}}^{2}}$, where wCV is the within‐subject coefficient of variation. Inter‐observer reproducibility was assessed using the intraclass correlation coefficient (ICC; two‐way random‐effects model, absolute agreement, single measurement) with 95% confidence intervals (CIs). The Wilcoxon rank sum test was used to compare 3D MRE parameters between the following groups: CLD participants versus healthy volunteers, protocols 1 versus 2, and no steatosis versus steatosis. Because fibrosis, inflammation, and steatosis scores are ordinal variables, Spearman's correlation coefficient (*r*_*s*_) was used to assess the correlation between histological scores and between MRE viscoelastic parameters and histological findings. To further assess the independent effects of histological findings and MRE protocols on each MRE parameter, multivariable linear regression analysis was performed.

When protocol differences significantly affected MRE parameters in the linear regression analysis, a logistic regression analysis was conducted using the affected parameter as the predictor and the protocol as the covariate. The logistic regression analysis aimed to determine whether the protocol differences influenced the performance of MRE parameters in predicting fibrosis or inflammation. When a significant protocol effect was identified, protocol‐adjusted logistic regression models were used to evaluate the diagnostic performance. The diagnostic performance of 3D MRE for staging fibrosis (≥ F2, ≥ F4, and ≥ F5) and grading inflammation (≥ I1, ≥ I4, and ≥ I8) was assessed with areas under the receiver operating characteristic curves (AUROCs). The AUROCs were compared across frequencies using an independent‐group design due to the partially paired participants.

All statistical analyses were performed with MATLAB (R2024a, MathWorks Inc., Natick, MA, USA) or SPSS (v.29, IBM Corp., Armonk, NY, USA). A *p* value of less than 0.05 was considered statistically significant.

## 3 Results

### 3.1 Study Participants

A flowchart of included CLD participants is shown in Figure 1b. Of the initial 85 CLD participants, six were excluded due to incomplete MRE examinations. Among the remaining participants, 15 were excluded from the analysis at all frequencies due to technical failures and failure to pass the quality assessment, and 10 were excluded at only one or two frequencies due to failure to pass the quality assessment. The total number of CLD participants included in the analysis was 64 (40 men, 24 women; median age [range], 58 [21–76] years) across the available frequencies.

A total of 21 healthy volunteers (eight men, 13 women; median age [range], 28 [21–57] years) were included in the analysis across frequencies. One healthy volunteer at 16.7 Hz and one at the second 33.4‐Hz scan were excluded due to insufficient MRE quality. For the test–retest repeatability assessment at 33.4 Hz, 20 healthy volunteers were included.

### 3.2 Clinical and Histological Parameters

**FIGURE 2:**
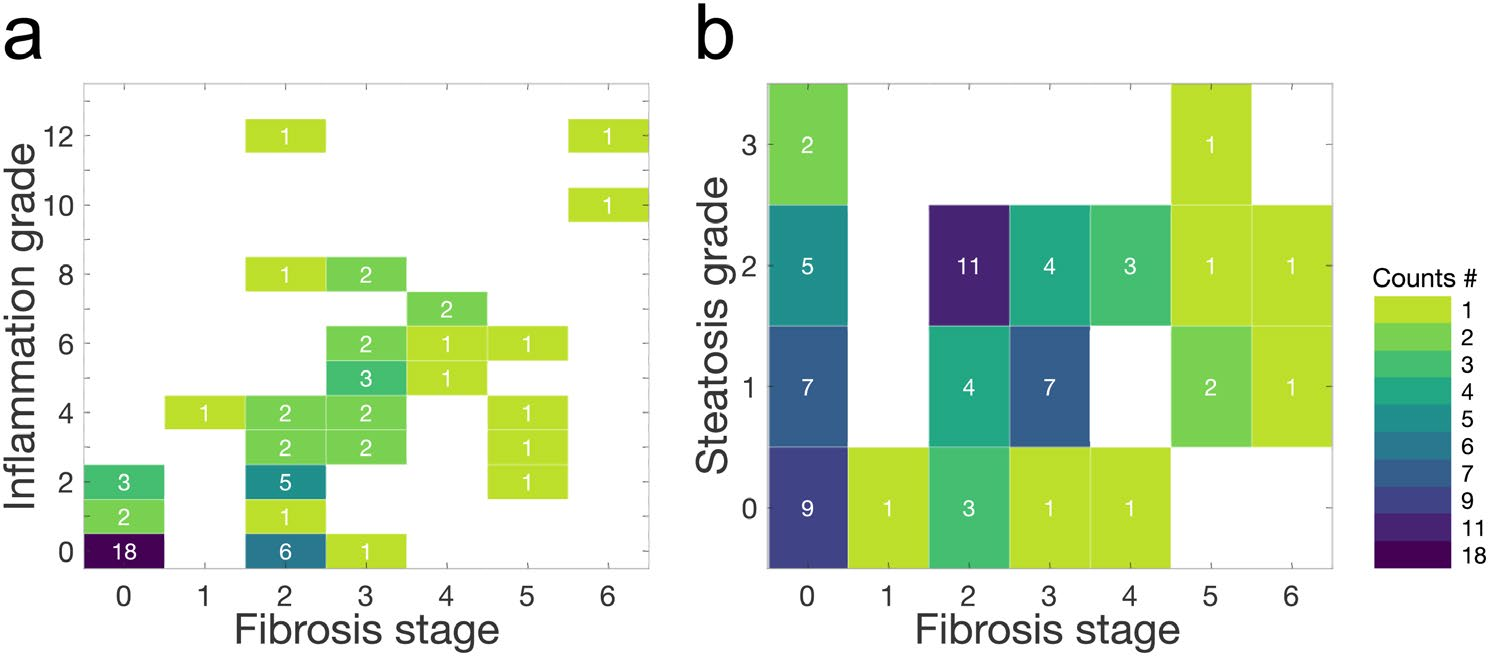
Distribution of histological findings using count heatmaps. (a) Distribution of fibrosis and inflammation. (b) Distribution of fibrosis and steatosis. Participant counts are annotated in each grid.

The demographic, laboratory, and histological data of the 64 CLD participants are presented in Table 1. The distribution of histological findings is shown in Figure 2. A significant correlation was observed between fibrosis stages and inflammation grades (*r*_*s*_ = 0.75) (Figure 2a). In CLD participants without fibrosis, 78.3% had an absence of inflammation, and 21.7% had mild inflammation (I1–2). At early fibrosis stages (F1–2), only 31.6% of CLD participants had an absence of inflammation. All CLD participants with F4 or higher had an inflammation grade of 2 or higher. There was no significant correlation between fibrosis and steatosis (*r*_*s*_ = 0.23, 95% confidence interval [CI] −0.02–0.46, *p* = 0.065) (Figure 2b), and CLD participants without fibrosis had varying steatosis grades (S0–3).

**TABLE 1:** Demographic, clinical, histopathologic characteristics, and diagnosis of the participants with CLD.

Characteristics	Value
Age (year)^a^	58.0 (50.0–68.5)
Sex	
Male	40 (62.5)
Female	24 (37.5)
BMI (kg/m^2^)^a^	29.5 (27.2–32.6)
Type 2 diabetes	20 (31.2)
Laboratory	
Platelet count (109/L)^b^	211.5 ± 58.7
Prothrombin time, INR^a^	1.0 (0.9–1.1)
Total bilirubin (μmol/L)^a^	10.0 (7.5–14.0)
AST (μkat/L)^a^	0.67 (0.53–0.97)
ALT (μkat/L)^a^	0.90 (0.59–1.40)
ALP (μkat/L)^a^	1.20 (0.98–1.60)
Histology	
Steatosis grade (Kleiner score)	
0	15 (23.4)
1	21 (32.8)
2	25 (39.1)
3	3 (4.7)
Fibrosis stage (Ishak score)	
0	23 (35.9)
1	1 (1.6)
2	18 (28.1)
3	12 (18.8)
4	4 (6.3)
5	4 (6.3)
6	2 (3.1)
Inflammation grade (Ishak score)	
0	25 (39.1)
1–3	17 (26.6)
4–7	16 (25.0)
8–12	6 (9.4)
Diagnosis	
Normal^c^	7 (10.9)
MASLD	32 (50.0)
ALD	4 (6.3)
MetALD	5 (7.8)
Others	16 (25.0)

*Note*: Categorical variables are presented as numbers with percentages in parentheses. Individual inflammation grades were 1 (*n* = 3), 2 (*n* = 9), 3 (*n* = 5), 4 (*n* = 6), 5 (*n* = 4), 6 (*n* = 4), 7 (*n* = 2), 8 (*n* = 3), 10 (*n* = 1), and 12 (*n* = 2). Other etiologies included autoimmune hepatitis (AIH) (*n* = 3), AIH and primary biliary cholangitis with lupus hepatitis (*n* = 1), drug‐induced liver injury (DILI) (*n* = 2), hemochromatosis (*n* = 1), MASLD with hemochromatosis (*n* = 3), MASLD with DILI (*n* = 2), ALD with hemochromatosis (*n* = 1), and nonspecific or unknown etiologies (*n* = 3). Abbreviations: ALD, alcohol‐related liver disease; ALP, alkaline phosphatase; ALT, alanine aminotransferase; AST, aspartate aminotransferase; BMI, body mass index; INR, international normalized ratio; MASLD, metabolic dysfunction‐associated steatotic liver disease; MetALD, metabolic dysfunction and alcohol‐related steatotic liver disease.

^a^ Continuous variables are shown as medians with IQRs in parentheses.

^b^ Continuous variables are shown as means ± SDs.

^c^ CLD participants whose liver biopsy showed normal histological features.

### 3.3 Repeatability and Comparison With Healthy Volunteers

The viscoelastic parameters, |G*|, *G′*, *G″*, and *ζ*, measured in the healthy volunteers for repeatability assessment are presented in Table 2. The RCs were lower for |G*| (16.76%) and *G′* (17.60%) than for *G″* (25.40%) and *ζ* (25.32%), showing better repeatability for |G*| and *G′*.

**TABLE 2:** MRE measurements on 20 healthy volunteers with test–retest repeatability assessment between scans at 33.4 Hz.

Parameter	Scan1 (mean ± SD)	Scan2 (mean ± SD)	RC (%)
Shear stiffness (|G*|), kPa	1.18 ± 0.19	1.13 ± 0.16	16.76
Storage modulus (*G′*), kPa	1.07 ± 0.16	1.02 ± 0.15	17.60
Loss modulus (*G″*), kPa	0.44 ± 0.08	0.41 ± 0.08	25.40
Damping ratio (*ζ*)	0.21 ± 0.02	0.21 ± 0.03	25.32

*Note*: One healthy volunteer was excluded due to insufficient MRE quality at the second scan. Abbreviations: RC, repeatability coefficient; SD, standard deviation.

The CLD participants' |G*|, *G′*, and *G″* were compared to those of healthy volunteers (Figure S1). At medium frequency, the CLD participants showed significantly higher |G*|, *G′*, and *G″* than the healthy volunteers with both protocols (Protocol 1, median [IQR]: 1.49 [1.26–1.67], 1.30 [1.14–1.49], and 0.58 [0.48–0.73] kPa; Protocol 2: 2.07 [1.67–2.35], 1.81 [1.50–2.00], and 0.73 [0.62–0.92] kPa; healthy volunteers: 1.19 [1.05–1.24], 1.04 [0.96–1.13], and 0.43 [0.39–0.49] kPa). At high frequency, CLD participants also showed significantly higher |G*|, *G′*, and *G″* with both protocols (Protocol 1: 1.88 [1.52–2.41], 1.72 [1.38–2.21], and 0.68 [0.60–0.87] kPa; Protocol 2: 2.41 [1.95–3.26], 2.25 [1.75–2.95], and 0.92 [0.74–1.13] kPa) compared with healthy volunteers (1.42 [1.33–1.53], 1.30 [1.23–1.39], and 0.52 [0.49–0.56] kPa). However, at the low frequency with Protocol 1, the CLD participants showed significantly lower |G*| (0.62 [0.51–0.71] vs. 0.76 [0.71–0.79] kPa) and *G′* (0.46 [0.36–0.51] vs. 0.62 [0.54–0.66] kPa), whereas *G″* did not differ (0.32 [0.29–0.39] vs. 0.36 [0.33–0.38] kPa, *p* = 0.096). No significant differences were observed for these MRE parameters with Protocol 2 (*p =* 0.227–0.650).

When the analysis was restricted to CLD participants with fibrosis, they still showed significantly lower |G*| and *G′* (Protocol 1, 0.60 [0.52–0.72] versus 0.76 [0.71–0.79] kPa; 0.42 [0.36–0.51] versus 0.62 [0.54–0.66] kPa) than healthy volunteers, and *G″* remained similar between groups (0.34 [0.29–0.40] versus 0.36 [0.33–0.38] kPa, *p* = 0.687). Based on these findings, further analyses focused on the medium and high frequency measurements. Distribution of low frequency measurements across histological scores is provided in Figure S2.

### 3.4 Inter‐Observer Reproducibility in CLD Participants

Inter‐observer reproducibility for MRE parameters was excellent, with ICC values ranging from 0.918 to 0.998 across frequencies (Table 3). The corresponding measurements obtained by the two observers are summarized in Table S2.

**TABLE 3:** Inter‐observer reproducibility for MRE viscoelastic parameters.

Parameter	Intraclass correlation coefficient (95% CI)		
	Low frequency (*n* = 58)	Medium frequency (*n* = 59)	High frequency (*n* = 61)
Shear stiffness (|G*|)	0.973 (0.827, 0.991)	0.991 (0.978, 0.995)	0.998 (0.997, 0.999)
Storage modulus (*G′*)	0.974 (0.862, 0.990)	0.987 (0.961, 0.994)	0.998 (0.996, 0.999)
Loss modulus (*G″*)	0.969 (0.818, 0.989)	0.991 (0.985, 0.995)	0.994 (0.990, 0.997)
Damping ratio (*ζ*)	0.925 (0.876, 0.955)	0.949 (0.877, 0.975)	0.918 (0.866, 0.950)

*Note: n* represents the number of participants with CLD. Abbreviation: CI, confidence interval.

### 3.5 Association Between MRE Parameters and Histological Findings

**FIGURE 3:**
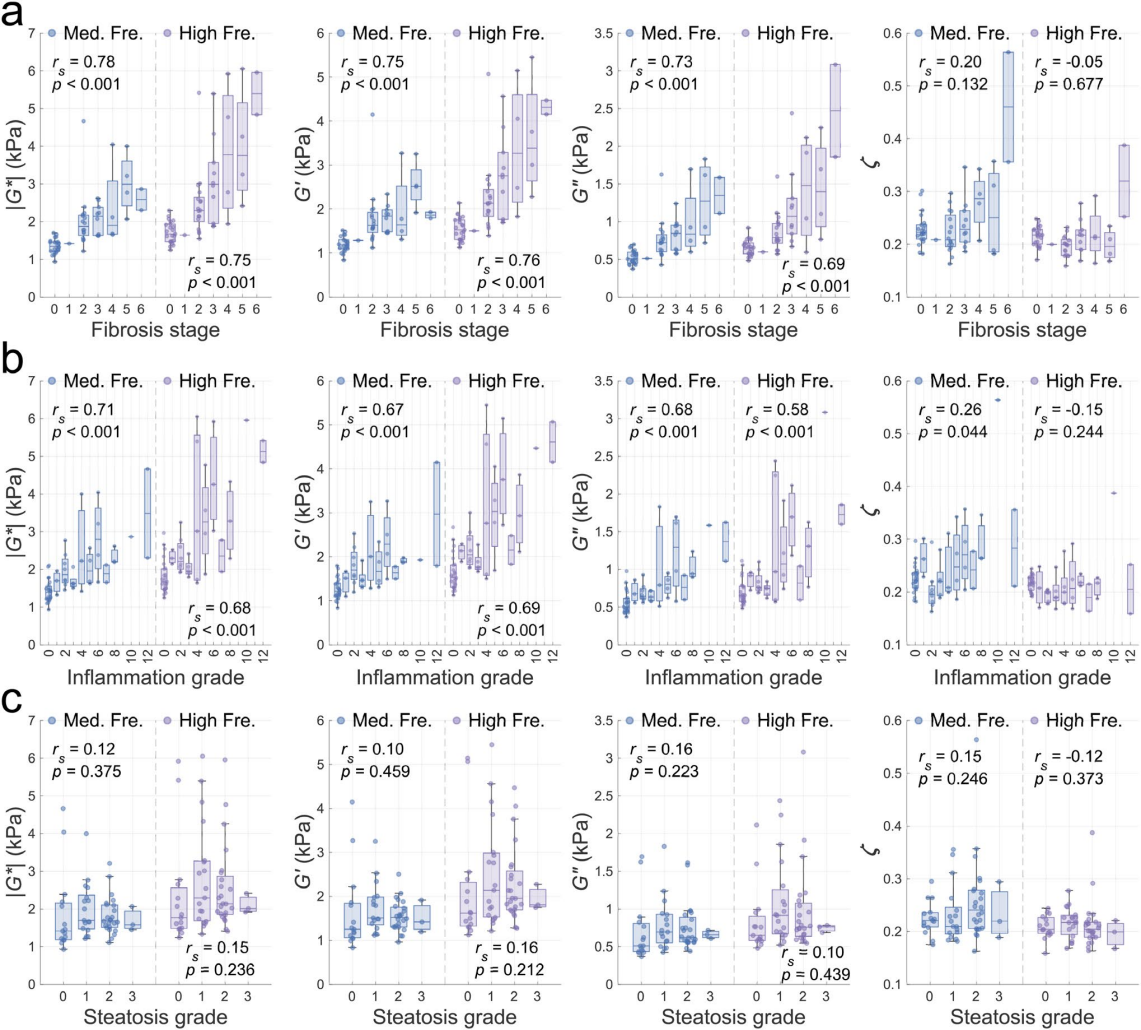
Distribution of MRE parameters across histological scores at medium and high frequencies. Scattered boxplots show shear stiffness (|G*|), storage modulus (*G′*), loss modulus (*G″*), and damping ratio (*ζ*) at two frequencies (medium in blue and high in purple) for each of the following histological categories: (a) fibrosis stage, (b) inflammation grade, and (c) steatosis grade. Significant Spearman's correlations (*r*_*s*_) were observed for |G*|, *G′*, and *G″* at both frequencies with fibrosis stage and inflammation grade, and for *ζ* at medium frequencies with inflammation grade. Dots show the individual data points. Med., medium; Fre., frequency.

**FIGURE 4:**
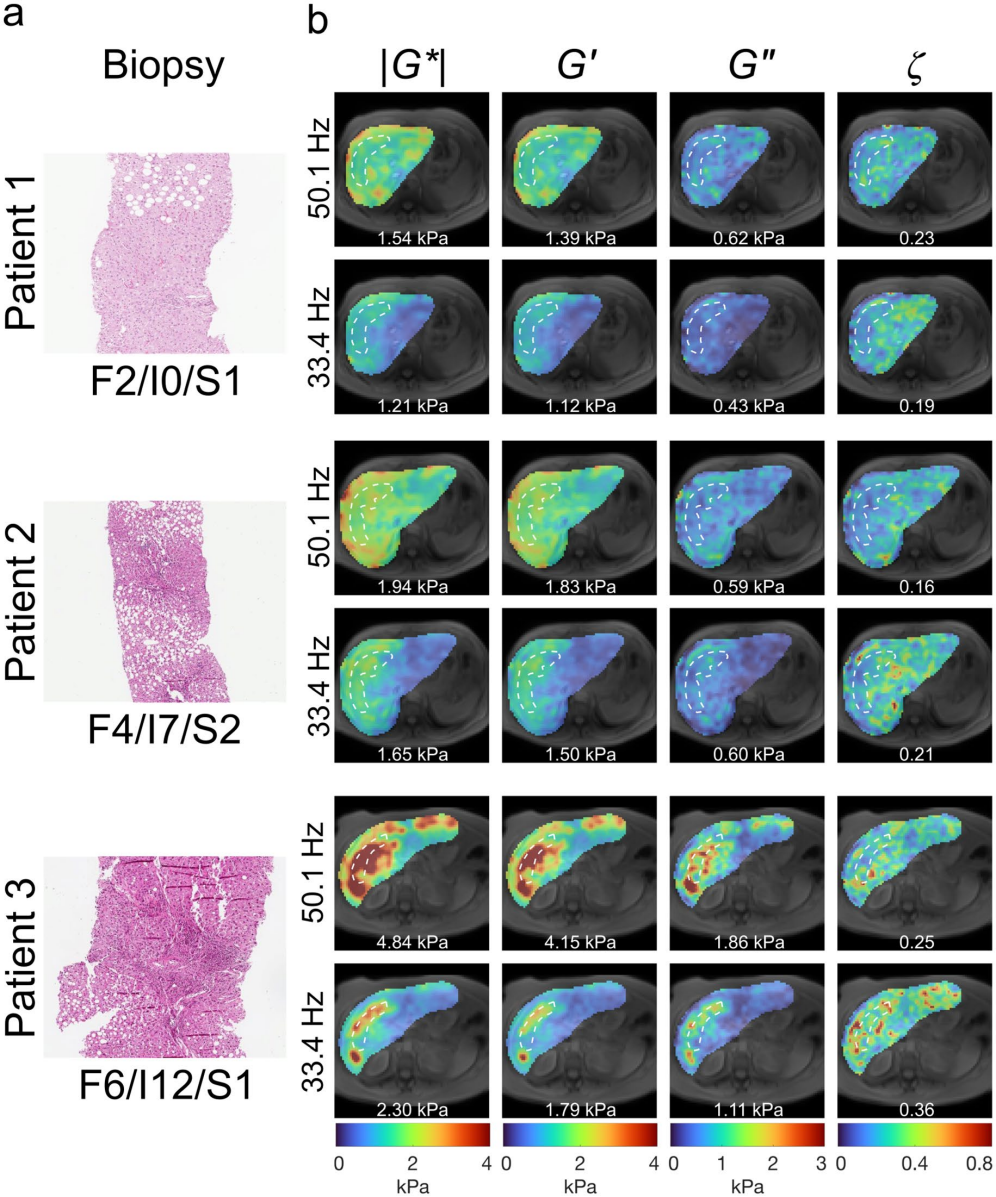
Examples of biopsy slices and MRE parametric maps for three CLD participants. Patient 1 (male, 55 years old, MASLD) with fibrosis stage 2, no inflammation and steatosis grade 1 (F2/I0/S1), patient 2 (male, 70 years old, MASLD) with fibrosis stage 4, inflammation grade 7 and steatosis grade 2 (F4/I7/S2), and patient 3 (female, 75 years old, MASLD and hemochromatosis) with fibrosis stage 6, inflammation grade 12 and steatosis grade 1 (F6/I12/S1). (a) Hematoxylin–eosin‐stained biopsy slices. (b) Corresponding parametric maps of shear stiffness (|G*|), storage modulus (*G′*), loss modulus (*G″*), and damping ratio (*ζ*) that were measured at medium (33.4 Hz) and high (50.1 Hz) driving frequencies. The reported values for each viscoelastic parameter (ROI shown in white dotted lines) are displayed beneath the corresponding maps.

Correlations between the viscoelastic parameters (|G*|, *G′*, *G″*, and *ζ*) and histological findings (fibrosis, inflammation, and steatosis) are summarized in Table 4 and Figure 3. Examples of histological specimens and MRE parametric maps are shown in Figure 4. In the comparison, |G*|, *G′* and *G″* at both medium and high frequencies showed significant correlations with fibrosis (*r*_*s*_ = 0.69–0.78) and inflammation (*r*_*s*_ = 0.58–0.71), with |G*| at the medium frequency having the strongest correlation with both fibrosis (*r*_*s*_ = 0.78) and inflammation (*r*_*s*_ = 0.71). No significant correlation was observed between fibrosis and *ζ* at either frequency (*p* = 0.132 and 0.677 for medium‐ and high frequency, respectively). Also, *ζ* gave a weak but significant correlation with inflammation at the medium frequency (*r*_*s*_ = 0.26), but not at the high frequency (*p* = 0.244). No MRE parameters showed a significant correlation with steatosis (*p* = 0.212–0.459).

**TABLE 4:** Spearman's correlation and multivariable linear regression between MRE viscoelastic parameters and fibrosis, inflammation, steatosis, and MRE protocols.

Parameter	Spearman's correlation		Multivariable regression analysis		
	*r*_*s*_ (95% CI)	*p*	*R* ^*2*^	*B* (95% CI)	*p*
Shear stiffness (|G*|)					
High frequency (*n* = 61)					
MRE protocol			0.58	‐0.02 (−0.47, 0.42)	0.928
Fibrosis	0.75 (0.61, 0.85)	**< 0.001**		0.37 (0.19, 0.55)	**< 0.001**
Inflammation	0.68 (0.52, 0.80)	**< 0.001**		0.14 (0.04, 0.23)	**0.006**
Steatosis	0.15 (−0.11, 0.40)	0.236		−0.19 (−0.45, 0.07)	0.143
Medium frequency (*n* = 59)					
MRE protocol			0.51	0.22 (−0.07, 0.50)	0.139
Fibrosis	0.78 (0.64, 0.86)	**< 0.001**		0.16 (0.04, 0.28)	**0.009**
Inflammation	0.71 (0.55, 0.82)	**< 0.001**		0.08 (0.02, 0.14)	**0.010**
Steatosis	0.12 (−0.15, 0.37)	0.375		−0.11 (−0.28, 0.05)	0.166
Storage modulus (G′)					
High frequency (*n* = 61)					
MRE protocol			0.58	0.08 (−0.30, 0.45)	0.690
Fibrosis	0.76 (0.62, 0.85)	**< 0.001**		0.30 (0.14, 0.45)	**< 0.001**
Inflammation	0.69 (0.53, 0.81)	**< 0.001**		0.12 (0.04, 0.20)	**0.005**
Steatosis	0.16 (−0.10, 0.40)	0.212		−0.15 (−0.37, 0.07)	0.165
Medium frequency (*n* = 59)					
MRE protocol			0.46	0.27 (0.02, 0.51)	**0.035**
Fibrosis	0.75 (0.61, 0.85)	**< 0.001**		0.11 (0.01, 0.22)	**0.027**
Inflammation	0.67 (0.49, 0.79)	**< 0.001**		0.06 (0.01, 0.11)	**0.026**
Steatosis	0.10 (−0.17, 0.35)	0.459		−0.09 (−0.23, 0.05)	0.180
Loss modulus (*G″*)					
High frequency (*n* = 61)					
MRE protocol			0.51	−0.09 (−0.29, 0.12)	0.400
Fibrosis	0.69 (0.52, 0.80)	**< 0.001**		0.17 (0.08, 0.25)	**< 0.001**
Inflammation	0.58 (0.38, 0.73)	**< 0.001**		0.04 (0.00, 0.09)	**0.047**
Steatosis	0.10 (−0.16, 0.35)	0.439		−0.08 (−0.20, 0.04)	0.184
Medium frequency (*n* = 59)					
MRE protocol			0.52	0.00 (−0.13, 0.14)	0.941
Fibrosis	0.73 (0.58, 0.83)	**< 0.001**		0.09 (0.03, 0.14)	**0.002**
Inflammation	0.68 (0.51, 0.80)	**< 0.001**		0.04 (0.01, 0.06)	**0.011**
Steatosis	0.16 (−0.11, 0.41)	0.223		−0.04 (−0.11, 0.04)	0.309
Damping Ratio (*ζ*)					
High frequency (*n* = 61)					
MRE protocol			0.08		
Fibrosis	−0.05 (−0.31, 0.21)	0.677			
Inflammation	−0.15 (−0.39, 0.11)	0.244			
Steatosis	−0.12 (−0.36, 0.15)	0.373			
Medium frequency (*n* = 59)					
MRE protocol			0.33	−0.04 (−0.07, −0.01)	**0.004**
Fibrosis	0.20 (−0.07, 0.44)	0.132		0.01 (−0.00, 0.02)	0.124
Inflammation	0.26 (0.00, 0.49)	**0.044**		0.01 (0.00, 0.01)	**0.022**
Steatosis	0.15 (−0.11, 0.40)	0.246		0.00 (−0.01, 0.02)	0.575

*Note: n* represents the number of participants with CLD. Adjusted *R* ^*2*^ values are provided. Significant *p* values (*p* < 0.05) are in bold. All *p* values of multivariable linear regression models were significant except for predicting the damping ratio at high frequency (*p* = 0.079). Abbreviations: CI, confidence interval; MRE, magnetic resonance elastography.

Multivariable linear regression analysis (Table 4) showed that both fibrosis and inflammation were significant covariates that contributed to |G*|, *G′*, and *G″* at both frequencies. In contrast, for *ζ*, the multivariable regression model did not yield a significant *R* ^*2*^ value at the high frequency (adjusted *R* ^*2*^ = 0.08, *p* = 0.079), while at the medium frequency, inflammation was significantly independently correlated with *ζ*. The two different MRE protocols showed no significant influence on the measurement of |G*| and *G″* at either frequency, or on *G′* at the high frequency (*p* = 0.139–0.941), but significantly affected both *G′* and *ζ* at the medium frequency.

### 3.6 Diagnostic Performance of MRE

The impact of protocol differences on *G′* and *ζ* at the medium frequency for predicting fibrosis and inflammation is summarized in Table S3. Protocol differences significantly affected only *ζ* for detecting inflammation (I ≥ 1) and predicting mild inflammation (I ≥ 4) (odds ratios, 7.70 and 8.96, respectively). The AUROCs of |G*|, *G′*, *G″*, and *ζ* in assessing fibrosis and inflammation are presented in Table 5, where protocol‐adjusted AUROCs are provided for *ζ* for I ≥ 1 and I ≥ 4. Cutoff values, sensitivity, specificity, and accuracy are provided in Table S4.

**TABLE 5:** Areas under the receiver operating curves (AUROCs) of the MRE parameters in staging fibrosis and grading inflammation.

Parameter	Fibrosis			Inflammation		
	F ≥ 2	F ≥ 4	F ≥ 5	I ≥ 1	I ≥ 4	I ≥ 8
Shear stiffness (|G*|)						
High frequency	0.91 (0.84, 0.98)	0.88 (0.76, 0.99)	0.90 (0.80, 1.00)	0.87 (0.78, 0.96)	0.86 (0.75, 0.97)	0.89 (0.77, 1.00)
Medium frequency	0.95 (0.90, 1.00)	0.85 (0.74, 0.97)	0.91 (0.82, 1.00)	0.88 (0.80, 0.97)	0.87 (0.77, 0.97)	0.90 (0.82, 0.98)
Storage modulus (*G′*)						
High frequency	0.92 (0.85, 0.98)	0.88 (0.78, 0.99)	0.90 (0.80, 1.00)	0.88 (0.79, 0.96)	0.86 (0.75, 0.97)	0.88 (0.76, 1.00)
Medium frequency	0.95 (0.90, 1.00)	0.82 (0.68, 0.95)	0.89 (0.80, 0.98)	0.87 (0.78, 0.96)	0.84 (0.73, 0.94)	0.84 (0.73, 0.95)
Loss modulus (*G″*)						
High frequency	0.87 (0.78, 0.96)	0.84 (0.66, 1.00)	0.88 (0.73, 1.00)	0.80 (0.69, 0.91)	0.82 (0.68, 0.96)	0.87 (0.73, 1.00)
Medium frequency	0.91 (0.84, 0.98)	0.86 (0.74, 0.98)	0.90 (0.80, 1.00)	0.85 (0.75, 0.95)	0.87 (0.76, 0.97)	0.92 (0.85, 0.99)
Damping Ratio (*ζ*)						
Medium frequency				0.76 (0.63, 0.89)^a^	0.82 (0.70, 0.94)^a^	0.83 (0.64, 1.00)

*Note*: Data in parentheses are 95% confidence intervals. *p* ≤ 0.001 for all AUROCs.

^a^ Adjusted for MRE protocol.

For staging fibrosis, |G*|, *G′*, and *G″* showed high AUROCs (0.82–0.95) with comparable performance between medium and high frequencies (*p* = 0.327–0.896, Table S5). For grading inflammation, |G*|, *G′*, and *G″* also yielded high AUROCs (0.80–0.92) with comparable performance between medium and high frequencies (*p* = 0.506–0.909, Table S5), while *ζ* showed moderate to good performance at the medium frequency (AUROC = 0.76–0.83).

### 3.7 Subgroup Analysis of Steatosis

**FIGURE 5:**
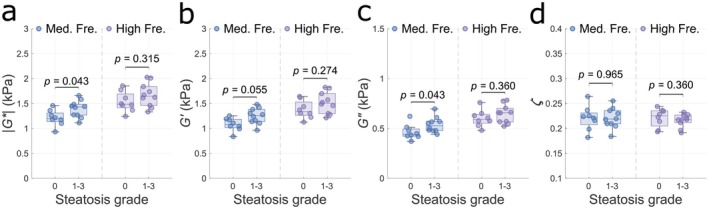
Comparison of MRE parameters by steatosis status in CLD participants without fibrosis or inflammation. Scattered boxplots show (a) shear stiffness (|G*|), (b) storage modulus (*G′*), (c) loss modulus (*G″*), and (d) damping ratio (*ζ*) at two frequencies (medium in blue and high in purple) stratified by steatosis. Comparison of MRE parameters between no steatosis and steatosis was performed using the Wilcoxon rank sum test. Exact *p* values were reported. Dots show the individual data points. Med., medium; Fre., frequency.

Steatosis subgroup analysis in CLD participants with no fibrosis and no inflammation (*n* = 18, characteristics are presented in Table S6) is shown in Figure 5. No significant differences in 3D MRE parameters were observed between the two MRE protocols (*p* = 0.203–1.000) (Figure S3). In the subgroup, at the medium frequency, |G*| and *G″* were significantly higher among the CLD participants with steatosis compared to those without (median [IQR]: 1.43 [1.27–1.48] vs. 1.20 [1.14–1.31] kPa; 0.53 [0.48–0.57] vs. 0.44 [0.42–0.49] kPa), while *G′* showed no significant difference (1.26 [1.14–1.38] vs. 1.08 [1.02–1.18] kPa, *p* = 0.055). At the high frequency, |G*|, *G′*, and *G″* exhibited no difference (1.65 [1.46–1.84] vs. 1.48 [1.41–1.69] kPa, *p* = 0.315; 1.51 [1.30–1.70] vs. 1.34 [1.26–1.53] kPa, *p* = 0.274; 0.66 [0.57–0.70] vs. 0.59 [0.55–0.64] kPa, *p* = 0.360). The value of *ζ* did not differ between CLD participants with steatosis and those without (0.22 [0.21–0.24] vs. 0.22 [0.21–0.23] kPa, *p* = 0.965 at medium; 0.22 [0.21–0.23] vs. 0.23 [0.20–0.24] kPa, *p* = 0.360 at high frequency).

## 4 Discussion

In this prospective study, we investigated the association between histology‐assessed hepatic inflammation, fibrosis, and steatosis with 3D‐MRE‐derived viscoelastic parameters at multiple frequencies in participants with CLD, with a primary focus on the role of 3D MRE in assessing inflammation. We did not observe a clear relationship between the damping ratio and inflammation at high frequencies. In contrast, at medium frequencies, the damping ratio showed a weak positive correlation with inflammation, and an independent association with it remained in the multivariable linear regression analysis.

The relationship between the damping ratio and hepatic inflammation remains inconsistent across previous studies. While some studies have reported an increase in the damping ratio with inflammation at the early disease stage [10, 14, 15], one study observed a decrease in patients with MASLD [13], and others have found it to be insensitive to inflammation [11, 25, 26]. These discrepancies can probably be explained by the wide differences in cohorts and MRE acquisition methods across studies. Despite these discrepancies, our findings suggest that the damping ratio has potential for independently characterizing inflammation at medium frequencies.

In our cohort, fibrosis and inflammation coexisted with a strong positive correlation. Our results suggest that both fibrosis and inflammation independently affected the measurement of MRE shear stiffness and the storage modulus. These findings align with previous studies suggesting that inflammation can lead to increased stiffness [11, 27, 28, 29, 30]. Additionally, fibrosis and inflammation independently influenced loss modulus in our cohort. Fibrosis has previously been shown to be an independent factor affecting loss modulus in both preclinical [31] and clinical CLD studies [16, 25]. Such an elevation of loss modulus with fibrosis can be explained by a possible mechanism in which not only a stiff, but also a viscous extracellular matrix, is deposited during the progression of fibrosis [32].

The calculated AUROCs demonstrated that all assessed MRE parameters, at both medium and high frequencies, showed good diagnostic performance for staging fibrosis and fair to good performance for grading inflammation. At medium frequencies, shear stiffness, storage modulus, and loss modulus showed comparable diagnostic performance for detecting fibrosis to those at high frequencies. Previous studies have reported the diagnostic accuracy of MRE for staging fibrosis to be equivalent across a frequency range of 45–62.5 Hz [33, 34]. Our results extend the lower boundary of this frequency range to 33.4 and 36 Hz.

When comparing different parameters for grading inflammation, the damping ratio at the medium frequency showed a slightly lower AUROC than other 3D MRE parameters. Notably, the damping ratio significantly correlated with inflammation, independent of fibrosis, unlike the shear stiffness, storage modulus, and loss modulus, which were also influenced by fibrosis. The higher AUROC values for these may be attributed to the strong correlation between fibrosis and inflammation in our cohort. The fair performance of the medium frequency‐derived damping ratio further supports its potential for the independent characterization of inflammation.

In this study, we also investigated the association between hepatic steatosis and 3D MRE viscoelastic parameters. Our results are consistent with previous publications concluding that biopsy‐conformed steatosis has no significant influence on either 2D MRE stiffness [26, 27, 35] or 3D MRE viscoelastic [15, 16, 26] measurements. In CLD participants without fibrosis or inflammation, higher shear stiffness and loss modulus were observed at medium frequency in those with steatosis. A similar increase in loss modulus has also been reported in rats with ‘simple steatosis’ [17]. These observations may suggest a possible frequency‐dependent sensitivity of viscoelastic parameters to early steatosis‐related mechanical changes.

Independent characterization of inflammation and fibrosis is clinically important for accurate assessment of disease activity in participants with CLD. Our study suggests the potential of medium frequency 3D MRE in this regard. The damping ratio at medium frequency was independently associated with inflammation and demonstrated moderate diagnostic performance for grading inflammation.

Furthermore, the fibrosis staging performance of 3D MRE was comparable between medium and high frequencies, making medium frequency a clinically favorable choice due to its improved sensitivity to inflammation without compromising fibrosis assessment. Although medium frequency 3D MRE also showed sensitivity to early steatosis changes in the absence of inflammation and fibrosis, steatosis did not confound the 3D MRE assessment of inflammation and fibrosis.

For future studies using multifrequency 3D MRE, it will be possible to explore the multifrequency dispersion coefficient (i.e., assuming an ideal spring‐pot power law model), which has been shown to better assess liver inflammation than single‐frequency‐derived viscoelastic parameters [18, 25]. The multifrequency dispersion coefficient was not included in our study due to concerns about the robustness of the low frequency MRE measurements in CLD participants. This limitation may be attributed to an improper ratio between mechanical wavelength and spatial resolution in the MRE acquisition setup. At lower frequencies, longer wavelengths can lead to oversampling (i.e., “too many” voxels per wavelength), causing underestimation of viscoelastic parameters, especially in patients with a stiffer liver [36]. One possible solution, recently proposed by Pagé et al. [37], is to retrospectively resample MRE data to achieve an optimal ratio between the wavelength and voxel size. Applying such an approach in future studies may help assure the reliability of low frequency MRE measurements and enable estimates of the multifrequency dispersion coefficient, for further exploration of its association with liver inflammation.

### 4.1 Limitations

First, this was a single‐center study with a fibrosis distribution skewed toward early stages and few participants with advanced fibrosis or cirrhosis. Etiology‐specific differences in viscoelastic parameters could not be robustly assessed due to the limited sample sizes within individual disease categories. The small number of CLD participants without fibrosis and inflammation limited the strength of the steatosis subgroup analysis. The overall cohort size was insufficient to support subgroup analyses by age, sex, and other clinical characteristics.

Second, fibrosis and inflammation were assessed using the Ishak scoring system, a widely used histopathological system for staging and grading CLD [38]. The Ishak system provides a comprehensive assessment with scores of 0–6 for fibrosis and 0–18 for inflammation. METAVIR is another commonly used system with a simpler framework (fibrosis 0–4; inflammation 0–3) that is easier to apply in routine clinical application [38]. Although good concordance between the Ishak and METAVIR systems has been reported [39], differences in scoring frameworks should be considered when comparing results across studies.

Third, this study lacked an independent test set. Cutoff values were data‐derived rather than established thresholds. The performance results should be considered exploratory and may be optimistically biased. The non‐identical participant groups across frequencies required an independent‐group design rather than a paired comparison for AUROC analyses, which may introduce selection bias and reduce statistical power.

Fourth, two MRE protocols with slightly different vibration frequencies were used due to a system upgrade. Pooling data acquired with the two protocols may introduce systematic differences, including frequency‐related differences in measured values, which may not be fully accounted for by regression adjustment. Test–retest repeatability was evaluated in healthy volunteers using the initial acquisition protocol. A repeatability assessment was not performed for the upgraded protocol, and the repeatability dataset was not acquired concurrently with CLD participant examinations, which may introduce potential temporal bias.

Finally, only two frequencies were analyzed after excluding low frequency data due to suspected spatial sampling artifacts, preventing estimation of the frequency dispersion coefficient. The 3D MRE research system requires separate post‐acquisition reconstruction, unlike commercial systems that provide immediate wave and confidence maps for quality checking. The absence of immediate evaluation resulted in some excluded datasets during post‐processing. Future integration of an immediate quality check into the scanner interface could reduce data loss.

## 5 Conclusion

The damping ratio derived using 3D MRE at medium frequency was independently associated with hepatic inflammation. The fibrosis staging performance of 3D MRE was comparable at medium and high frequencies. Steatosis had a limited effect on viscoelastic parameters and did not confound the assessment of inflammation and fibrosis. These findings show the potential of medium frequency 3D MRE to improve sensitivity to inflammation while maintaining accurate fibrosis assessment in a clinically relevant cohort.

## Funding

This work was supported by Vinnova (Sweden's Innovation Agency), the Swedish Research Council for Engineering Sciences and Natural Sciences (VR/NT), 2020‐04826, and ALF funding (Avtal om Läkarutbildning och Forskning; Agreement on Medical Education and Research) from Region Östergötland (Östergötland County Council).

## Conflicts of Interest

The authors declare no conflicts of interest.

## Supporting information


**Table S1:** Detailed MRE acquisition parameters.
**Table S2:** MRE viscoelastic values for inter‐observer reproducibility assessment.
**Table S3:** Odds ratios and *p* values of damping ratio (*ζ*) and storage modulus (*G′*) at medium frequencies from the logistic regression analysis for grading inflammation and staging fibrosis. MRE protocol is included as a covariate assessed in each model.
**Table S4:** Cutoff values, sensitivity, specificity, and accuracy of MRE parameters in staging fibrosis and grading inflammation.
**Table S5:** *P* values of the AUROCs compared between medium and high frequencies.
**Table S6:** Demographic and clinical characteristics of the subgroup cohort (*n* = 18) without fibrosis and without inflammation.
**Figure S1:** Comparison of MRE measurements between patients and healthy volunteers. Scattered boxplots showing (a) shear stiffness (|G*|), (b) storage modulus (*G′*), and (c) loss modulus (*G″*) at three frequencies. Magenta represents patients, and green represents healthy volunteers. Dots show the individual data points. Med., medium; Fre., frequency.
**Figure S2:** Distribution of low frequency MRE parameters across histological scores. Plots show shear stiffness (|G*|), storage modulus (*G′*), loss modulus (*G″*), and damping ratio (*ζ*) in relation to (a) fibrosis stage, (b) inflammation grade, and (c) steatosis grade. Spearman's correlation coefficients (*r*_*s*_) and *p* values are shown in each plot. Only |G*| and *G″* exhibited weak correlations with fibrosis, while other MRE parameters showed no correlation with fibrosis, inflammation, or steatosis. Dots show individual data points.
**Figure S3:** Comparison of MRE parameters between protocols in patients without fibrosis or inflammation. Scattered boxplots show (a) shear stiffness (|G*|), (b) storage modulus (*G′*), (c) loss modulus (*G″*), and (d) damping ratio (*ζ*) at two frequencies (medium in blue and high in purple) stratified by MRE protocols. MRE parameters did not differ between protocols 1 and 2. Comparisons between the two groups were performed using the Wilcoxon rank sum test. Exact *p* values were reported. Dots show individual data points. Med., medium; Fre., frequency.
