# Supplementary material for: Chronic Liver Disease: Assessing Inflammation and Fibrosis Using Three‐Dimensional MR Elastography With Same‐Day Biopsy in a Prospective Cohort
Source: J Magn Reson Imaging. 2026 Apr 2;64(1):306–19. doi: 10.1002/jmri.70319 (PMC13254032; doi:10.1002/jmri.70319)
Supplement: Supplementary file 1 — Table S1: Detailed MRE acquisition parameters. Table S2: MRE viscoelastic values for inter‐observer reproducibility assessment. Table S3: Odds ratios and p values of damping ratio (ζ) and storage modulus (G′) at medium frequencies from the logistic regression analysis for grading inflammation and staging fibrosis. MRE protocol is included as a covariate assessed in each model. Table S4: Cutoff values, sensitivity, specificity, and accuracy of MRE parameters in staging fibrosis and grading inflammation. Table S5: P values of the AUROCs compared between medium and high frequencies. Table S6: Demographic and clinical characteristics of the subgroup cohort (n = 18) without fibrosis and without inflammation. Figure S1: Comparison of MRE measurements between patients and healthy volunteers. Scattered boxplots showing (a) shear stiffness (|G*|), (b) storage modulus (G′), and (c) loss modulus (G″) at three frequencies. Magenta represents patients, and green represents healthy volunteers. Dots show the individual data points. Med., medium; Fre., frequency. Figure S2: Distribution of low frequency MRE parameters across histological scores. Plots show shear stiffness (|G*|), storage modulus (G′), loss modulus (G″), and damping ratio (ζ) in relation to (a) fibrosis stage, (b) inflammation grade, and (c) steatosis grade. Spearman's correlation coefficients (r s ) and p values are shown in each plot. Only |G*| and G″ exhibited weak correlations with fibrosis, while other MRE parameters showed no correlation with fibrosis, inflammation, or steatosis. Dots show individual data points. Figure S3: Comparison of MRE parameters between protocols in patients without fibrosis or inflammation. Scattered boxplots show (a) shear stiffness (|G*|), (b) storage modulus (G′), (c) loss modulus (G″), and (d) damping ratio (ζ) at two frequencies (medium in blue and high in purple) stratified by MRE protocols. MRE parameters did not differ between protocols 1 and 2. Comparisons between the two grou [file JMRI-64-306-s001.docx]

**Supplementary Material**

**Table S1.** Detailed MRE acquisition parameters.

| Acquisition parameter | Protocol 1 | Protocol 2 |
| --- | --- | --- |
| Driving frequency (Hz) | 16.7/33.4/50.1 | 18/36/54 |
| Field-of-view (mm^2^) | 320 × 320 | 320 × 320 |
| Reconstructed matrix | 80 × 80 | 80 × 80 |
| Flip angle (degree) | 15 | 15 |
| Echo time (ms) | 4.61 | 6.91 |
| Repetition time (ms) | 64.8 | 125/118/78.7 |
| # of breath holds × breath hold time (s) | 4 × 17 | 4 × 18.7/17.7/19.7 |
| # of slices | 9 | 8 |
| Slice thickness (mm) | 4 | 4 |
| # of phase offsets | 12 | 4 |
| # of image shots per vibration | 9 | 4/2/2 |
| Motion encoding directions | P, M, S, R | P, M, S, R |

Abbreviations: M, frequency encoding; P, phase encoding; R, reference without motion encoding; S, slice selection.

**Table S2.** MRE viscoelastic values for inter-observer reproducibility assessment.

| Parameter | Low frequency  (*n* = 58) | Medium frequency  (*n* = 59) | High frequency  (*n* = 61) |
| --- | --- | --- | --- |
| Shear stiffness (*\|G*\|*), kPa |  |  |  |
| Observer 1 | 0.65 ± 0.23 0.63 (0.51–0.76) | 1.88 ± 0.74 1.66 (1.43–2.18) | 2.56 ± 1.26 2.07 (1.72–2.95) |
| Observer 2 | 0.62 ± 0.22 0.59 (0.47–0.73) | 1.83 ± 0.70 1.67 (1.37–2.12) | 2.56 ± 1.24 2.05 (1.74–2.92) |
| Storage modulus (*G′*), kPa |  |  |  |
| Observer 1 | 0.48 ± 0.17 0.47 (0.36–0.56) | 1.63 ± 0.60 1.48 (1.24–1.88) | 2.29 ± 1.07 1.89 (1.57–2.69) |
| Observer 2 | 0.45 ± 0.17 0.45 (0.34–0.53) | 1.58 ± 0.57 1.47 (1.17–1.80) | 2.29 ± 1.03 1.85 (1.58–2.65) |
| Loss modulus (*G″*), kPa |  |  |  |
| Observer 1 | 0.34 ± 0.12 0.33 (0.27–0.41) | 0.75 ± 0.34 0.63 (0.55–0.86) | 0.96 ± 0.53 0.76 (0.63–1.05) |
| Observer 2 | 0.32 ± 0.12 0.31 (0.24–0.38) | 0.75 ± 0.32 0.64 (0.55–0.82) | 0.98 ± 0.54 0.77 (0.64–1.06) |
| Damping ratio (*ζ*), kPa |  |  |  |
| Observer 1 | 0.43 ± 0.08 0.42 (0.36–0.47) | 0.24 ± 0.06 0.22 (0.20–0.26) | 0.21 ± 0.03 0.21 (0.19–0.23) |
| Observer 2 | 0.42 ± 0.08 0.43 (0.36–0.48) | 0.25 ± 0.06 0.23 (0.21–0.27) | 0.22 ± 0.04 0.22 (0.19–0.23) |
| ROI size, cm^3^ |  |  |  |
| Observer 1 | 43.33 ± 18.38 42.37 (31.55–54.98) | 42.92 ± 16.55 41.86 (29.57–52.86) | 40.80 ± 15.76 37.18 (31.60–51.02) |
| Observer 2 | 56.68 ± 22.86 59.14 (40.19–70.59) | 56.33 ± 21.32 57.66 (43.44–69.50) | 51.23 ± 19.62 48.19 (37.09–65.46) |

*Note*, *n* represents the number of patients. Variables are shown as mean ± standard deviation and median with interquartile range in parentheses.

Abbreviation: CI, confidence interval; ROI, region of interest.

**Table S3.** Odds ratios and *p*-values of damping ratio (*ζ*) and storage modulus (*G′*) at medium frequencies from the logistic regression analysis for grading inflammation and staging fibrosis. MRE protocol is included as a covariate assessed in each model.

| Parameter | Odds ratio | *p-*value |
| --- | --- | --- |
| Inflammation |  |  |
| I ≥ 1 |  |  |
| MRE protocol | 7.70 | **0.002** |
| Damping ratio (*ζ*) | 4.45E+07 | **0.028** |
| I ≥ 4 |  |  |
| MRE protocol | 8.96 | **0.008** |
| Damping ratio (ζ) | 9.96E+12 | **0.002** |
| I ≥ 8 |  |  |
| MRE protocol | 9.54 | 0.082 |
| Damping ratio (ζ) | 1.71E+11 | **0.009** |
| I ≥ 1 |  |  |
| MRE protocol | 1.30 | 0.726 |
| Storage modulus (G′) | 148.02 | **< 0.001** |
| I ≥ 4 |  |  |
| MRE protocol | 0.91 | 0.900 |
| Storage modulus (G′) | 23.04 | **0.003** |
| I ≥ 8 |  |  |
| MRE protocol | 1.32 | 0.785 |
| Storage modulus (G′) | 3.81 | **0.037** |
| Fibrosis |  |  |
| F ≥ 2 |  |  |
| MRE protocol | 1.04 | 0.971 |
| Storage modulus (G′) | 3.02E+04 | **< 0.001** |
| F ≥ 4 |  |  |
| MRE protocol | 0.18 | 0.093 |
| Storage modulus (G′) | 9.85 | **0.008** |
| F ≥ 5 |  |  |
| MRE protocol | 1.24 | 0.835 |
| Storage modulus (G′) | 4.37 | **0.026** |

*Note:* Significant *p*-values (*p* < 0.05) are in bold.

Abbreviations: MRE, magnetic resonance elastography

**Table S4.** Cutoff values, sensitivity, specificity, and accuracy of MRE parameters in staging fibrosis and grading inflammation.

| Parameters | Fibrosis | | | Inflammation | | |
| --- | --- | --- | --- | --- | --- | --- |
|  | F ≥ 2 | F ≥ 4 | F ≥ 5 | I ≥ 1 | I ≥ 4 | I ≥ 8 |
| Shear stiffness (*\|G*\|*) |  |  |  |  |  |  |
| High frequency |  |  |  |  |  |  |
| Cutoff value (kPa) | 2.29 | 2.38 | 3.13 | 1.86 | 2.78 | 3.26 |
| Sensitivity | 0.68 (0.51, 0.80) [25/37] | 0.90 (0.60, 0.98) [9/10] | 0.83 (0.44, 0.97) [5/6] | 0.92 (0.78, 0.97) [33/36] | 0.75 (0.53, 0.89) [15/20] | 0.83 (0.44, 0.97) [5/6] |
| Specificity | 1.00 (0.86, 1.00) [24/24] | 0.73 (0.59, 0.83) [37/51] | 0.87 (0.76, 0.94) [48/55] | 0.72 (0.52, 0.86) [18/25] | 0.95 (0.84, 0.99) [39/41] | 0.89 (0.78, 0.95) [49/55] |
| Accuracy | 0.80 (0.69, 0.88) [49/61] | 0.75 (0.63, 0.84) [46/61] | 0.87 (0.76, 0.93) [53/61] | 0.84 (0.72, 0.91) [51/61] | 0.89 (0.78, 0.94) [54/61] | 0.89 (0.78, 0.94) [54/61] |
| Medium frequency |  |  |  |  |  |  |
| Cutoff value (kPa) | 1.61 | 1.64 | 2.03 | 1.59 | 2.11 | 2.17 |
| Sensitivity | 0.89 (0.75, 0.96) [32/36] | 1.00 (0.72, 1.00) [10/10] | 1.00 (0.61, 1.00) [6/6] | 0.86 (0.71, 0.94) [30/35] | 0.74 (0.51, 0.88) [14/19] | 1.00 (0.61, 1.00) [6/6] |
| Specificity | 0.91 (0.73, 0.98) [21/23] | 0.57 (0.43, 0.70) [28/49] | 0.74 (0.60, 0.84) [39/53] | 0.79 (0.60, 0.91) [19/24] | 0.93 (0.80, 0.97) [37/40] | 0.83 (0.71, 0.91) [44/53] |
| Accuracy | 0.90 (0.80, 0.95) [53/59] | 0.64 (0.52, 0.75) [38/59] | 0.76 (0.64, 0.85) [45/59] | 0.83 (0.72, 0.91) [49/59] | 0.86 (0.75, 0.93) [51/59] | 0.85 (0.73, 0.92) [50/59] |
| Storage modulus (*G′*) |  |  |  |  |  |  |
| High frequency |  |  |  |  |  |  |
| Cutoff value (kPa) | 1.92 | 2.26 | 2.97 | 1.65 | 2.44 | 2.86 |
| Sensitivity | 0.76 (0.60, 0.87) [28/37] | 0.90 (0.60, 0.98) [9/10] | 0.83 (0.44, 0.97) [5/6] | 0.94 (0.82, 0.98) [34/36] | 0.75 (0.53, 0.89) [15/20] | 0.83 (0.44, 0.97) [5/6] |
| Specificity | 0.96 (0.80, 0.99) [23/24] | 0.75 (0.61, 0.84) [38/51] | 0.89 (0.78, 0.95) [49/55] | 0.68 (0.48, 0.83) [17/25] | 0.93 (0.81, 0.97) [38/41] | 0.87 (0.76, 0.94) [48/55] |
| Accuracy | 0.84 (0.72, 0.91) [51/61] | 0.77 (0.65, 0.86) [47/61] | 0.89 (0.78, 0.94) [54/61] | 0.84 (0.72, 0.91) [51/61] | 0.87 (0.76, 0.93) [53/61] | 0.87 (0.76, 0.93) [53/61] |
| Medium frequency |  |  |  |  |  |  |
| Cutoff value (kPa) | 1.44 | 1.75 | 1.78 | 1.44 | 1.75 | 1.78 |
| Sensitivity | 0.86 (0.71, 0.94) [31/36] | 0.80 (0.49, 0.94) [8/10] | 1.00 (0.61, 1.00) [6/6] | 0.80 (0.64, 0.90) [28/35] | 0.74 (0.51, 0.88) [14/19] | 1.00 (0.61, 1.00) [6/6] |
| Specificity | 0.91 (0.73, 0.98) [21/23] | 0.76 (0.62, 0.85) [37/49] | 0.75 (0.62, 0.85) [40/53] | 0.79 (0.60, 0.91) [19/24] | 0.85 (0.71, 0.93) [34/40] | 0.75 (0.62, 0.85) [40/53] |
| Accuracy | 0.88 (0.77, 0.94) [52/59] | 0.76 (0.64, 0.85) [45/59] | 0.78 (0.66, 0.87) [46/59] | 0.80 (0.68, 0.88) [47/59] | 0.81 (0.70, 0.89) [48/59] | 0.78 (0.66, 0.87) [46/59] |
| Loss modulus (*G″*) |  |  |  |  |  |  |
| High frequency |  |  |  |  |  |  |
| Cutoff value (kPa) | 0.79 | 1.02 | 1.08 | 0.67 | 0.92 | 1.26 |
| Sensitivity | 0.68 (0.51, 0.80) [25/37] | 0.80 (0.49, 0.94) [8/10] | 0.83 (0.44, 0.97) [5/6] | 0.89 (0.75, 0.96) [32/36] | 0.75 (0.53, 0.89) [15/20] | 0.83 (0.44, 0.97) [5/6] |
| Specificity | 0.96 (0.80, 0.99) [23/24] | 0.84 (0.72, 0.92) [43/51] | 0.84 (0.72, 0.91) [46/55] | 0.64 (0.45, 0.80) [16/25] | 0.85 (0.72, 0.93) [35/41] | 0.91 (0.80, 0.96) [50/55] |
| Accuracy | 0.79 (0.67, 0.87) [48/61] | 0.84 (0.72, 0.91) [51/61] | 0.84 (0.72, 0.91) [51/61] | 0.79 (0.67, 0.87) [48/61] | 0.82 (0.71, 0.90) [50/61] | 0.90 (0.80, 0.95) [55/61] |
| Medium frequency |  |  |  |  |  |  |
| Cutoff value (kPa) | 0.71 | 0.72 | 0.92 | 0.57 | 0.74 | 0.88 |
| Sensitivity | 0.69 (0.53, 0.82) [25/36] | 0.90 (0.60, 0.98) [9/10] | 0.83 (0.44, 0.97) [5/6] | 0.91 (0.78, 0.97) [32/35] | 0.79 (0.57, 0.91) [15/19] | 1.00 (0.61, 1.00) [6/6] |
| Specificity | 1.00 (0.86, 1.00) [23/23] | 0.71 (0.58, 0.82) [35/49] | 0.87 (0.75, 0.93) [46/53] | 0.71 (0.51, 0.85) [17/24] | 0.85 (0.71, 0.93) [34/40] | 0.85 (0.73, 0.92) [45/53] |
| Accuracy | 0.81 (0.70, 0.89) [48/59] | 0.75 (0.62, 0.84) [44/59] | 0.86 (0.75, 0.93) [51/59] | 0.83 (0.72, 0.91) [49/59] | 0.83 (0.72, 0.91) [49/59] | 0.86 (0.75, 0.93) [51/59] |
| Damping Ratio (*ζ*) |  |  |  |  |  |  |
| Medium frequency |  |  |  |  |  |  |
| Cutoff value |  |  |  |  |  | 0.26 |
| Sensitivity |  |  |  | 0.83 (0.67, 0.92) [29/35] ^†^ | 0.84 (0.62, 0.94) [16/19] ^†^ | 0.83 (0.44, 0.97) [5/6] |
| Specificity |  |  |  | 0.71 (0.51, 0.85) [17/24] ^†^ | 0.70 (0.55, 0.82) [28/40] ^†^ | 0.79 (0.67, 0.88) [42/53] |
| Accuracy |  |  |  | 0.78 (0.66, 0.87) [46/59] ^†^ | 0.75 (0.62, 0.84) [44/59] ^†^ | 0.80 (0.68, 0.88) [47/59] |

*Note:* Data in parentheses are 95% confidence intervals, with the numbers of subjects in brackets. The optimal cutoff values were determined by the Youden index.

^†^Adjusted for MRE protocol.

**Table S5.** *P*-values of the AUROCs compared between medium and high frequencies.

| Parameter | Fibrosis | | | Inflammation | | |
| --- | --- | --- | --- | --- | --- | --- |
|  | F ≥ 2 | F ≥ 4 | F ≥ 5 | I ≥ 1 | I ≥ 4 | I ≥ 8 |
| Shear stiffness (*\|G*\|*) | 0.327 | 0.755 | 0.896 | 0.833 | 0.909 | 0.907 |
| Storage modulus (*G′*) | 0.444 | 0.421 | 0.881 | 0.920 | 0.755 | 0.747 |
| Loss modulus (*G″*) | 0.466 | 0.799 | 0.758 | 0.506 | 0.586 | 0.684 |

**Table S6.** Demographic and clinical characteristics of the subgroup cohort (*n* = 18) without fibrosis and without inflammation.

| Characteristic | Value |
| --- | --- |
| Age (year)^*^ | 49.6 ± 17.2 |
| Sex |  |
| Male | 12 (66.7%) |
| Female | 6 (33.3%) |
| BMI (kg/m^2^)^*^ | 27.5 ± 4.2 |
| Type 2 diabetes | 3 (16.7%) |
| Laboratory |  |
| Platelet count (10^9^/L)^*^ | 225.2 ± 60.7 |
| Prothrombin time, INR^†^ | 0.95 (0.90–1.00) |
| Total bilirubin (µmol/L)^*^ | 9.9 ± 4.3 |
| AST (µkat/L)^†^ | 0.59 (0.45–0.67) |
| ALT (µkat/L)^†^ | 0.73 (0.40–0.96) |
| ALP (µkat/L)^†^ | 1.15 (1.00–1.20) |
| Histology |  |
| Steatosis grade |  |
| 0 | 8 (44.4%) |
| 1 | 5 (27.8%) |
| 2 | 4 (22.2%) |
| 3 | 1 (5.6%) |
| Diagnosis |  |
| Normal^‡^ | 7 (38.9%) |
| MASLD | 9 (50.0%) |
| MetALD | 1 (5.6%) |
| Hemochromatosis | 1 (5.6%) |

Abbreviations: ALP, alkaline phosphatase; ALT, alanine aminotransferase; AST, aspartate aminotransferase; BMI, body mass index; INR, international normalised ratio; MASLD, metabolic dysfunction-associated steatotic liver disease; MetALD, metabolic dysfunction and alcohol-related steatotic liver disease.

^*^Continuous variables are shown as means ± SDs.

^†^Continuous variables are shown as medians with IQRs in parentheses.

^‡^Patients whose liver biopsy showed normal histological features.

**
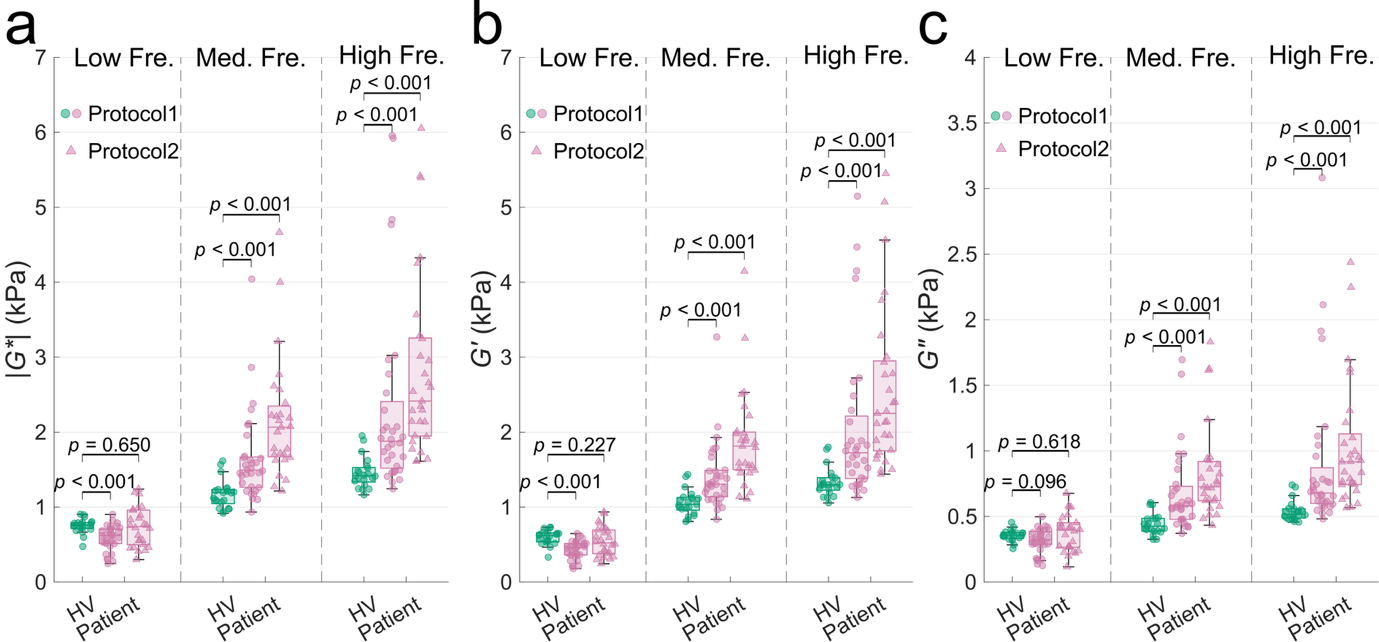
**

**Figure S1.** Comparison of MRE measurements between patients and healthy volunteers. Scattered boxplots showing (a) shear stiffness (*|G*|*)*,* (b) storage modulus (*G′*), and (c) loss modulus (*G″*) at three frequencies. Magenta represents patients, and green represents healthy volunteers. Dots show the individual data points. Med., medium; Fre., frequency.

**
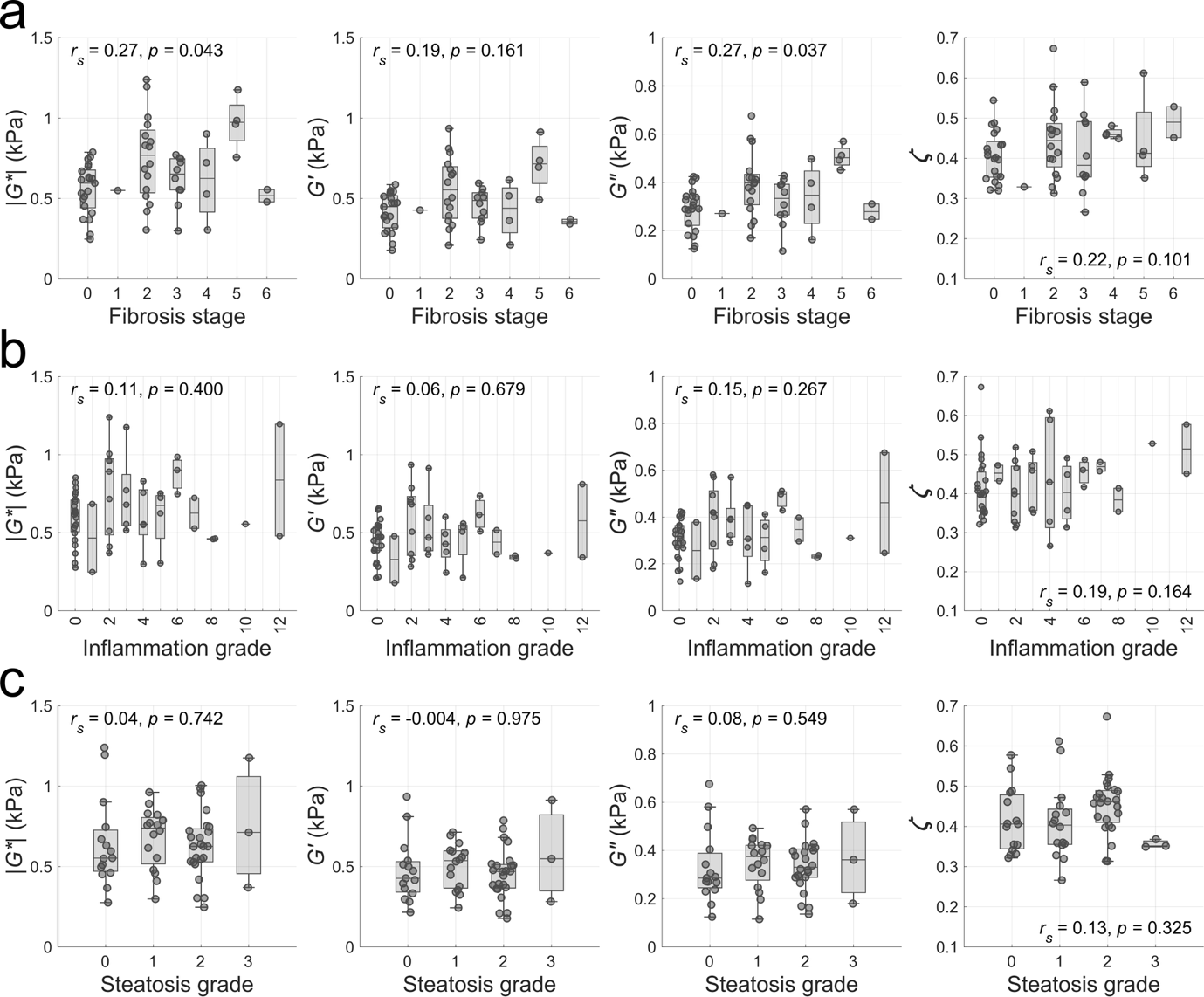
**

**Figure S2.** Distribution of low-frequency MRE parameters across histological scores. Plots show shear stiffness (|*G*|*), storage modulus (*G′*), loss modulus (*G″*), and damping ratio (*ζ*) in relation to (a) fibrosis stage, (b) inflammation grade, and (c) steatosis grade. Spearman’s correlation coefficients (*r_s_*) and *p*-values are shown in each plot. Only *|G*|* and *G″* exhibited weak correlations with fibrosis, while other MRE parameters showed no correlation with fibrosis, inflammation, or steatosis. Dots show individual data points.

**
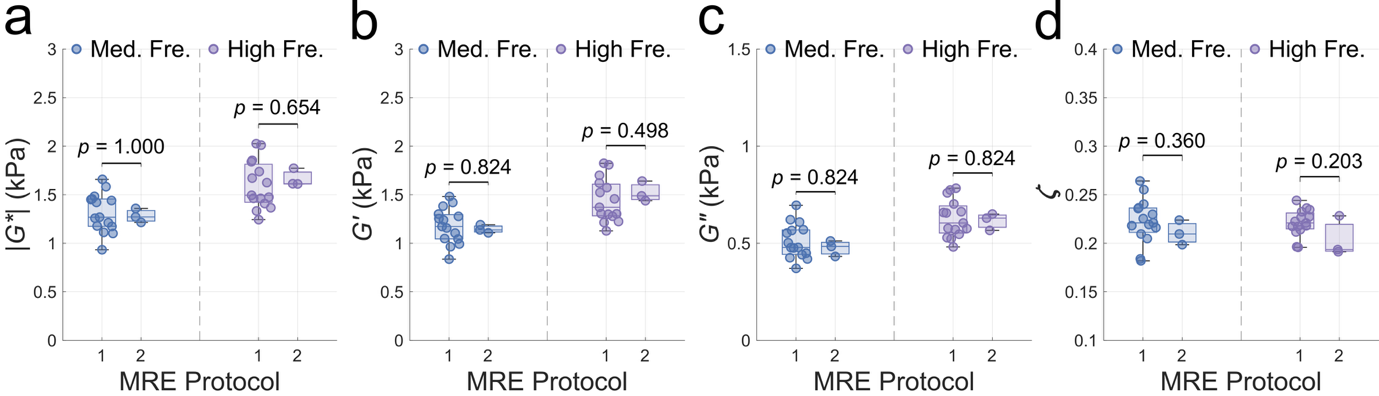
**

**Figure S3.** Comparison of MRE parameters between protocols in patients without fibrosis or inflammation. Scattered boxplots show (a) shear stiffness (|*G*|*), (b) storage modulus (*G′*), (c) loss modulus (*G″*), and (d) damping ratio (*ζ*) at two frequencies (medium in blue and high in purple) stratified by MRE protocols. MRE parameters did not differ between protocols 1 and 2. Comparisons between the two groups were performed using the Wilcoxon rank sum test. Exact *p*-values were reported. Dots show individual data points. Med., medium; Fre., frequency.
